# Health, priority to the worse off, and time

**DOI:** 10.1007/s11019-018-9825-2

**Published:** 2018-01-19

**Authors:** Anders Herlitz

**Affiliations:** 1 Department of Philosophy, Linguistics and Theory of Science, University of Gothenburg, Box 200, 405 30 Gothenburg, Sweden; 2 Department of Global Health and Population, Harvard T.H. Chan School of Public Health, Boston, USA

**Keywords:** Priority setting, Prioritarianism, Time, Political philosophy, Ethics

## Abstract

It is a common view that benefits to the worse off should be given priority when health benefits are distributed. This paper addresses how to understand who is worse off in this context when individuals are differently well off at different times. The paper argues that the view that this judgment about who is worse off should be based solely on how well off individuals are when their complete lives are considered (i.e. ‘the complete lives view’) is implausible in this context. Instead, it is argued that a pluralistic stance toward this issue should be accepted. This pluralistic stance recognizes that also the view that only focuses on how well off individuals are now and in the future (i.e. ‘the forward-looking view’) is relevant. The argument is based on appeals to intuitive judgments concerning who is worse off in different cases and reference to various underlying reasons why priority to benefits to the worse off is justified.

It is widely acknowledged in the literature on health-related priority-setting that benefits to the worse off should be given some priority (e.g. Brock 2001; Dolan and Tsuchiya 2012; Eyal et al. 2013; Herlitz 2017a, b; Herlitz and Horan 2016; Nord 2005; Ottersen 2013; Sen 2001; Williams 1997). Spelling out exactly what this means is, however, a complicated matter for a variety of reasons. There are many dimensions in which someone can be worse off (e.g., in terms of wellbeing, health, opportunities, resources), and there are many ways to give priority to someone (e.g., by giving extra weight to their claims, lexical priority to their claims, or by earmarking a fixed amount of resources for their claims). Furthermore, there are many different reasons *why* one might want to give priority to benefits to the worse off: is it because it is good to promote equality for its own sake, good to promote equality for other reasons, because benefits to the worse off matter more, because the worse off typically fall under some sufficiency threshold, or for many of these (and maybe other) reasons (cf. Adler 2012; Brock 2001; Crisp 2002, 2003; Eyal et al. 2013; Herlitz 2017a, b; Herlitz and Horan 2016; Ottersen et al. 2014; Segall 2016, Chap. 8; Temkin 2003)?

In this paper, I will sidestep many of these questions and focus on a different issue that must be dealt with by a comprehensive approach to how to establish who is worse off, namely how to think about the fact that individuals might be differently well off in terms of what matters at different times. Someone with relatively good health at the present might have endured significant ill health in the past, and someone with relatively large health needs in the present might have relatively good health outlooks. How should inequalities of this kind be dealt with when individuals are ranked with the purpose of ascribing priority to health benefits to the worse off? I will argue against the sometimes suggested view which I will call ‘the complete lives view’ toward this issue, i.e. the view that the *only* thing that matters when we establish who is worse off is how well off individuals are in terms of the unit of measurement over their complete lives (cf. Adler 2012; Hirose 2005; Holtug 2010, chs. 8–10; Lippert-Rasmussen 2003; McKerlie 1989; Segall 2016; Temkin 1993). I will argue that although there certainly is *something* to the complete lives view in this context, there are other aspects that matter, and these might point in a different direction. To deal with this, I suggest that we should accept a pluralist view; both the complete lives view and the ‘forward-looking view’ (i.e. the view that ignores the past) matter when individuals are ranked with the purpose of giving priority to benefits to the worse off. Besides being a theoretically important issue, this has vast potential practical implications for health policy. It influences how to think about and value past ill health when scarce resources are distributed, and gives policy makers reasons to put greater (but not complete) emphasis on mitigating health inequalities in the future rather than focusing on compensating individuals who have been badly off in the past.

Before engaging with the argument, one significant difference between the literature on distributive ethics (which has explored these questions in quite some length) and the literature on population-level bioethics and health economics (which is somewhat less advanced in this area) should be pointed out. Whereas it in the general ethics literature is common to discuss equality, priority and the goodness of different distributions in terms of the distribution of quality of life, welfare or wellbeing in a very general sense (e.g., Lippert-Rasmussen 2003; Parfit 1997; Segall 2016; Temkin 1993, 2003), the literature in population-level bioethics and health economics that focuses on health inequality, equity and regrettable inequality in health often addresses the distribution of *health* and/or other *health-related* benefits (e.g., opportunity for health, social determinants of health) (e.g., Eyal et al. 2013; Hausman 2007, 2013; Nord 2005; Ottersen 2013; Williams 1997). Thus, whereas debates in moral philosophy often focus on the distribution of general ‘welfare’, ‘wellbeing’ or ‘benefits’ (e.g., Fleurbaey et al. 2009; Segall 2016; Temkin 1993), health economists and population-level bioethicists often use summary measures of health such as Quality-Adjusted Life Years (QALY) in order to study distributions (e.g., Dolan and Tsuchiya 2012; Herlitz and Horan 2016; Nord 2005; Ottersen et al. 2014; Williams 1997).

This difference is important because ‘welfare’ and ‘wellbeing’ might have different properties than summary measure of health such as QALY, and these differences can have a significant importance for how the worse off should be identified when the distribution over time differs (cf. Herlitz 2018a). Notably, whereas health is almost always taken to be something that can be treated as separable when health states at different times are aggregated, it is not at all obvious that general quality of life, welfare or wellbeing can be treated in this way (cf. Adler 2012; Broome 1991, Chap. 11; Herlitz 2018a; Hirose 2005; Mirrlees 1982; Strotz 1955–1956). For example, if someone who reaches the age of 80 enjoys 35 QALYs during her first 40 years and 20 QALYs during her following 40 years, she will have enjoyed 55 QALYs during her lifetime. A simple summation of 35 and 20 establishes this. In more formal terms, approaches to distribution of health typically rely on assumptions of *additivity* (lifetime unit measurements of health are summations of sublifetime attributes) and *atomism* (lifetime unit measurements of health are determined independently of other features of the population). Contrary to this, if someone who reaches the age of 80 enjoys 35 units of wellbeing during her first 40 years and 20 units of wellbeing during her following 40 years, it is an open question whether her lifetime wellbeing should be seen as equivalent to 55 units of wellbeing or not (cf. Adler 2012, Chap. 6; Herlitz 2018a). This is related to substantive, and highly contested, views regarding the nature of wellbeing. In other words, it is an open question whether additivity and atomism should be accepted or not when wellbeing is the unit of measurement. Indeed, as I will return to later in this paper, there are some very good reasons not to accept that wellbeing has these properties (cf. Adler 2012, Chap. 6; Herlitz 2018a).

Throughout, I will assume that one is interested in establishing who is worse off *in terms of health-related quality of life*, and not overall wellbeing (cf. Dolan and Tsuchiya 2012; Herlitz and Horan 2016; Nord 2005; Ottersen 2013; Ottersen et al. 2014). A lot can be said about this assumption and about approaches to allocation of health-related resources that rely on this assumption. First, it is not obvious how to establish what aspects of a life are ‘health-related’. Is it anything that can affect someone’s health, or is the concept more narrow? To operationalize this concept, one must establish which resources are health-related and which are not (cf. Segall 2007; Wilson 2009). Second, it is not obvious why we should focus only on this. Based on the idea that there are different ‘spheres of justice’, some might for example hold that distribution of health is the only thing that matters when health-related resources are allocated (cf. Brock 2003; Walzer 1983). This would make the assumption quite reasonable. On the other hand, and as many moral and political philosophers engaging with this debate have pointed out, although tremendously important, inequalities in health are not the *only* thing that matters when the goodness of different outcomes is assessed (e.g. Broome 1988; Hausman 2007, 2013, 2015; Herlitz 2017b; Temkin 2013, 2014).

I will not take a position on these issues here, and I make the assumption for reasons that are unrelated to these debates. I introduce the assumption because I am particularly interested in discussing theories and suggestions that rely on the assumption and that advocate the use of this sort of approach, for example “Lifetime QALY prioritarianism in priority setting” (Ottersen et al. 2014). Some of the arguments that follow presuppose that one approaches inequalities and health in this way, but not all of them do. In particular, it is the first and the last argument in the third section (“Theoretical Justifications”) that make use of the distinction between health and overall wellbeing. I believe that the general argument that is presented has some force also in contexts where the focus is ‘welfare’ or ‘wellbeing’, but the argument is stronger in contexts where the focus is some summary measure of health that relies on assumptions of additivity and atomism (or more broadly: any context in which the unit of measurement allows for additivity and atomism).

The paper is structured in the following way. In the first section, I introduce the complete lives view and point to some of its strengths. In the second section, I question whether this can provide rankings that are in tune with our intuitions, and claim that it cannot. This provides a reason to accept a more nuanced approach to how to think about who is worse off. I suggest that what I call the ‘forward-looking view’ reflects widely held intuitions concerning who is worse off. In the third section, I provide two theoretical justifications for the forward-looking view. In the fourth section, I discuss how one can combine the forward-looking view and the complete lives view. I end the paper with some concluding remarks.

## The complete lives view

Discussions in distributive ethics that address how to specify who is worse off when people are differently well off at different times originally focused on how to value equality (cf. Hirose 2005; Lippert-Rasmussen 2003; McKerlie 1989; Temkin 1993). Expressed as a specification of an egalitarian view, the complete lives view states that: “different people’s shares of [goods] should be equal when we consider the total amounts of those things that they receive over the complete course of their lives (McKerlie 1989, p. 476).” This can be transformed into a specification of the related question concerning who ought to have priority based on the fact that she is worse off:

[Someone] may be worse off than others in terms of her complete life, considering her life as a whole in the temporal sense and comparing it to the complete lives of others. [The complete lives view] is that having a worse life in this way entitles someone to priority (McKerlie 1997, p. 288).

The complete lives view is popular in the theoretical ethics literature on the value of equality as well as in much of the literature on so-called prioritarianism (i.e. the view that benefits to the worse off matter more) (cf. Adler 2012; Dworkin 1981; Hirose 2015; Holtug 2010, chs. 8–10; Kappel 1997; Nagel 1979, 1991; Rawls 1971; Parfit 1997; Segall 2016, Chap. 3). It is also very influential in the literature that directly deals with equity in health and priority to the worse off in terms of health when health-related resources are allocated (cf. Dolan and Tsuchiya 2012; Herlitz 2018a; Nord 2005; Norheim and Asada 2009; Ottersen 2013; Ottersen et al. 2014; Williams 1997; WHO 2014). There are also good reasons to focus on how well off people are over their whole lives. Focusing on how well off people are over their whole lives reflects concerns for what has been called ‘separateness of persons’ (cf. Rawls 1971; Segall 2016), it seems to be a prerequisite for taking individual responsibility into account, and it has been suggested to reflect concerns for ‘distributive fairness’ more broadly (cf. Bidadanure 2016).

One early expression of the complete lives view with respect to health is found in Alan Williams’s endorsement of the view that the notion of ‘being worse off’ in terms of health is related to not having a ‘fair inning’, i.e. a fair/sufficient amount of health over a life (Williams 1997; Nord 2005, 2013). More recently, Trygve Ottersen, Ottar Maestad and Ole Frithjof Norhem have suggested that:

According to one reasonable specification, the worse off are those with the fewer lifetime QALYs [i.e. Quality-Adjusted Life Years, a common summary measure of health], i.e., those who will have the fewer QALYs over their entire lifespan. This comprehensive specification incorporates both quality and quantity of health as well as past, present and future health (Ottersen et al. 2014, p. 2).

For yet another example, consider a (admittedly somewhat vague^1^) passage from a recent report from the World Health Organization’s (WHO) on equity and priority setting on the path to universal health coverage:

When focusing on health […] it is important to focus not only on those that *currently* have the worst health. Indeed, there are good reasons to start with those worse off over their *lifetime*. There is both empirical and theoretical support for why one should focus on those worse off thus understood, rather than those worse off here and now or the worse off only prospectively (WHO 2014, p. 15).

As is clear from this passage from the WHO’s report, the complete lives view is often promoted in opposition to the view that only focuses on who *currently* is worse off. Some inequality, and some rankings of who are worse off, is indeed often established by looking at a specific period of time such as the present. For example, it is common among economists to compare income and wealth distributions within specific temporal segments such as calendar years (e.g. Atkinson 2015; Sen and Foster 1997). There are also approaches to inequality in health that at least on the surface appear to apply a narrow focus and only address ill health in specific temporal segments (typically the present). For example, a recent study by Raj Chetty et al. looks at the association between income and life expectancy in the United States between 2001 and 2014 (Chetty et al. 2016). In this important study, they use data on income and life expectancy at particular years, and point to health inequalities in these temporal segments. Thereby, past ill health (as well as past income) is completely ignored. Furthermore, by focusing on life expectancy and ignoring the quality of health in the future, it is questionable to say the least whether they manage to take future health into account. Others, such as the Swedish Parliamentary Priorities Commission, directly emphasize the importance of giving priority to individuals who currently suffer from ill health (SOU 1995). Such a practice ignores both past ill health and ill health in the future. In the United Kingdom, the National Institute for Health and Care Excellence (NICE) has recently recognized (although not implemented in their cost-effectiveness models) the importance of giving priority to the worse off, which they assess by looking at absolute and relative shortfalls from normal healthy life expectancy (Cookson 2015). Also this type of approach clearly ignores past ill health.

In light of the widespread practice of focusing only on certain periods of time, shifting toward the complete lives view seems attractive. If the purpose truly is to give priority to the worse off in terms of health, the complete lives view is appealing. If we, following much practice in health economics (cf. Cookson 2015; Dolan and Tsuchiya 2012; Hausman 2015; Herlitz and Horan 2016) represent health-related quality of life on a scale 0 (for dead) and 1 (for full health) and let these be invariable within the temporal segments (i.e. each individual has the same health-related quality of life throughout the temporal segments), it seems like Oscar in the following outcome is worse off than Jerry, and benefits to Oscar should intuitively get priority [I follow a convention in moral philosophy and use two-person cases in order to make the exposition simpler, but the argument is equally applicable to inequalities between groups (cf. Hirose 2005; McKerlie 1989; Lippert-Rasmussen 2003; Segall 2016; Temkin 1993)]:

**Table:** 

Case 1					
Years	1–20	21–40	*t*_*0*_	41–60	61–80
Oscar	0.5	0.5	|	0.6	0.6
Jerry	1	1	|	0.6	0.6

It is clear that Oscar is worse off than Jerry when their whole lives are compared. In terms of QALY, Oscar enjoys 0.5 × 20 + 0.5 × 20 + 0.6 × 20 + 0.6 × 20 = 42 QALYs, while Jerry enjoys 1 × 20 + 1 × 20 + 0.6 × 20 + 0.6 × 20 = 64 QALYs. Jerry enjoys the equivalent of 22 years of life at perfect health more than Oscar. Clearly, Jerry is *much* better off than Oscar.

In case a social planner intervenes at *t*_*0*_, they have, on the complete lives view, some reason to give priority to benefits to Oscar since he is worse off, even if they both happen to be equally badly off in terms of health in light of only the present and/or the future. This seems to be in tune with our considered judgments in this case and it seems fair in case we connect fairness to separateness of persons (cf. Bidadanure 2016). Oscar really seems to be worse off than Jerry even from the perspective that they are currently both 41 years old and both of their current health-related quality of life is 0.6; how well off they have been so to speak tips the balance in favor of giving priority to benefits to Oscar.

Discussions around cases like these reveal how it is not only some part of their life that matters when two individuals are ranked in terms of how well off they are. It is a mistake to only focus on how well off someone currently is, or how well off they will be given their current health prospects. The complete lives view helps us see this.

## The forward-looking view

The complete lives view is appealing in that it broadens our focus and encourages us to not only look at how well off different people are in the present when we establish who is worse off. Yet, by treating each temporal segment equally I believe that it fails to capture *all* that matters when we establish who is worse off in these contexts. In this section I will introduce some cases that are largely inspired by Dennis McKerlie’s work on inequality, priority and time to make this case (McKerlie 1989, 1997, 2012). The purpose of these cases is to illustrate that the complete lives view has counterintuitive implications, and the argumentative technique largely relies on reference to intuitions. In the following section, I present two theoretical arguments for the conclusion drawn here. Consider the following outcome. Again, let the numbers represent health-related quality of life and assume that this is invariable for each person throughout each time period:

**Table:** 

Case 2					
Years	1–20	21–40	*t*_*0*_	41–60	61–80
Sarah	0.3	0.3	|	0.9	0.8
Theresa	1	1	|	0.3	0.3

It is clear that the complete lives view here suggests that Sarah is worse off than Theresa. Furthermore, from a perfectly neutral standpoint, in which we so to speak exist outside of time, it seems clear that Sarah really is worse off than Theresa. In terms of QALY, Sarah enjoys 0.3 × 20 + 0.3 × 20 + 0.9 × 20 + 0.8 × 20 = 46 QALYs, while Theresa enjoys 1 × 20 + 1 × 20 + 0.3 × 20 + 0.3 × 20 = 52 QALYs. Theresa enjoys the equivalent of 6 years of life at perfect health more than Sarah. Clearly, Theresa is much better off than Sarah.

Yet, what if we shift perspective? What if we take a (somewhat) more realistic point of view, and consider the following question: at the time when both Sarah and Theresa turn 41 years old, and Sarah’s health-related quality of life is 0.9 and Theresa’s health-related quality of life is 0.3, and when it is known that Sarah will have significantly better health than Theresa throughout the remainder of their lives, who is worse off and should be given priority? Sarah can look forward to the equivalent of 34 years of life at perfect health, while Theresa has the equivalent of a mere 12 years of life at perfect health in front of her.

Here, I contend that although we know that Sarah has endured much health-related problems in the past, while Theresa has been perfectly healthy up till now, Theresa should be considered to be worse off than Sarah, and priority should be given to benefits to Theresa. Of the following two outcomes, I think that Outcome 2.2 is better:

**Table:** 

Outcome 2.1					
Years	1–20	21–40	*t*_*0*_	41–60	61–80
Sarah	0.3	0.3	|	1	0.8
Theresa	1	1	|	0.3	0.3

**Table:** 

Outcome 2.2					
Years	1–20	21–40	*t*_*0*_	41–60	61–80
Sarah	0.3	0.3	|	0.9	0.8
Theresa	1	1	|	0.4	0.3

To reach this judgment, we must abandon the complete lives view that relies on summations of sublifetime attributes and introduce some other view. One view that can make sense of this intuition is what I will call ‘the forward-looking view’: someone may be worse off than others in terms of their current and future health, considering what is left of their life and comparing it to what is left of the life of others. The forward-looking view is that having a worse life in this way entitles someone to priority. Rather than comparing how well off people are over their whole lives, this view compares how well off people are at simultaneous temporal segments, now and in the future (i.e. it is a version of what McKerlie calls the ‘simultaneous segments view’ that only looks at current and future temporal segments, McKerlie 1989). This view focuses on how well off the different people are now, as well as how well off they will be in the future.

However, contrary to those who seem to favor an approach that *only* focuses on health prospects, I believe that it is also a mistake to completely ignore the past (cf. Ottersen 2013; Nord 2005); Consider Case 1 from the previous section gain:

**Table:** 

Case 1					
Years	1–20	21–40	*t*_*0*_	41–60	61–80
Oscar	0.5	0.5	|	0.6	0.6
Jerry	1	1	|	0.6	0.6

From the perspective of the complete lives view, it is obvious that Oscar is worse off than Jerry, and it is also clear that this would be our considered judgment from a temporally neutral perspective. In terms of QALY, Oscar enjoys 42 QALYs while Jerry enjoys 64 QALYs. Jerry enjoys the equivalent of 22 years of life at perfect health more than Oscar. Clearly, Jerry is much better off than Oscar.

Yet, if we adopt the forward-looking view and imagine an intervention at *t*_*0*_, Jerry and Oscar are equally badly off since they have equally poor prospects. They both have the same expected amount of QALY to enjoy, 0.6 × 20 + 0.6 × 20 = 24. On the forward-looking view, it follows that we should be indifferent toward the following two outcomes:

**Table:** 

Outcome 1.1					
Years	1–20	21–40	*t*_*0*_	41–60	61–80
Oscar	0.5	0.5	|	0.7	0.6
Jerry	1	1	|	0.6	0.6

**Table:** 

Outcome 1.2					
Years	1–20	21–40	*t*_*0*_	41–60	61–80
Oscar	0.5	0.5	|	0.6	0.6
Jerry	1	1	|	0.7	0.6

This seems wrong. Outcome 1.1 is clearly better than Outcome 1.2 in light of the intuitions we have concerning giving priority to the worse off. Oscar really should be considered to be worse off than Jerry at *t*_*0*_.

It seems, in other words, as if *both* the complete lives view and the forward-looking view get something right. One explanation for this might be that Shlomi Segall is right when he argues that *both* a prioritarianism that holds that benefits to those who are worse off at a specific moment matter more and an egalitarianism that holds that complete lives inequalities are bad must be part of the complete moral picture (cf. Kappel 1997; Segall 2016, Chap. 7). Such a theory might explain the judgments above. In some cases, the complete lives egalitarianism is the driving force of our judgments (e.g. Case 1) whereas in some cases priority to benefits to the worse off in a specific moment is the driving force of our judgments (e.g. Case 2). Another explanation might be that we have reason to care about both simultaneous segment and complete lives inequality (cf. Bidadanure 2016; McKerlie 1989; Temkin 1993).

It might be objected that, in *some* sense, the forward-looking view is already accounted for in the complete lives view. After all, future ill health is part of complete lives ill health. This is, I think, a very shallow way of understanding this argument, and in case one wants to defend the complete lives view in this way one needs to specify and adjust the complete lives view accordingly (i.e. allow it to give different weights to health states at different times). Rather, the discussion above reveals that our intuitions support the position that the present and the future *matter more* than the past, and health levels in the present and the future should not be aggregated in the same way as health levels in the past when we establish who is worse off. In the next section, I will present some theoretical justifications for accepting the forward-looking view.

## Theoretical justifications

In a paper that argues against what he calls ‘exclusion of past health’ (i.e. the view that only health prospects matter when individuals are ranked in terms of health for priority-setting), Trygve Ottersen claims that in the debate on these issues, the burden of proof falls upon those defending excluding past health (Ottersen 2013). It is somewhat misleading to call normative arguments ‘proofs’, but the question is warranted: What might the theoretical justification for taking a forward-looking perspective be? Although I believe that both the forward-looking view and the complete lives view must be adopted, the question of what justifies independent use of (or extra weight to) the forward-looking view must be addressed. In this section, I will present two different arguments in favor of this. I will argue that the forward-looking view can be justified with reference to (1) how inequality in status goods is bad; and (2) how the complete lives view toward inequality in wellbeing might in fact justify a forward-looking view toward how to establish who is worse off in terms of health.

### Differences in status goods

One reason why some inequalities are bad is that they can constitute, enable and lead to differences in status goods. Equality (and by inference priority to the worse off) is good not only because it is fair, but because it secures that certain goods are universally distributed (cf. Anderson 1999; Bidadanure 2016; Nagel 1979; Parfit 1997; O’Neill 2008; Rawls 2001; Scanlon 2003). On this view, it is not inequality as such that is bad, but rather the effects of inequality; equality is *instrumentally* valuable. Martin O’Neill presents a list of no less than six different reasons for why inequality might be bad in this way: (a) alleviation of inequality is often a requirement for the reduction of suffering; (b) inequality creates stigmatizing differences in status; (c) inequality creates objectionable relations of power; (d) inequality weakens self-respect; (e) inequality creates servility and deferential behavior; (f) inequality undermines fraternal relations (O’Neill 2008).

O’Neill does not address inequalities at different temporal segments in his paper, and he does not speak about inequalities in health. However, it seems clear that these worries primarily relate to situations in which individuals are differently well off at the same time, and not to whether the individuals end up having different amounts of goods when we compare their complete lives. The fact that two individuals might be equally well off on the complete lives view does not facilitate alleviation of suffering in case there are great inequalities in certain temporal segments, complete lives equality does not help against stigmatizing differences in status, undesirable power relations occur when people are differently well off at certain times, self-respect relates to how well off others are at the same time, as does fraternal relations. Consider a simple illustration: The fact that a slave and his owner switch places half-way through their lives so that they end up being equally well off on the complete lives view is not likely to remove stigmatizing differences in status, undesirable power relations, lack of self-respect and lack of fraternal relations. The type of equality that is beneficial because it promotes universal distribution of certain goods is simultaneous segments equality, not complete lives equality.

Although O’Neill does not address inequalities in *health*, I believe that the argument can be extended also to inequalities in health. At the surface, it looks as if the reasons O’Neill lists are primarily related to economic and political equality. Such inequalities clearly affect the distribution of status goods, power relations, self-respect and fraternal relations. Yet, so do inequalities in health. Firstly, inequalities in health are clearly related to inequalities in economic and political equality. Having better health gives clear economic and political advantages. Secondly, inequalities in health can more directly lead to differences in status goods, undesirable power relations, differences in self-respect as well as difficulties establishing fraternal relations. The fact that those with ill health often depend on assistance from people with better health clearly creates a power relation. Not being able to engage in the same sort of activities as one’s peers due to ill health might undermine self-respect. Certain fraternal relations in our world seem to be built around activities that those with ill health cannot participate in.

Giving priority to benefits to the person with relatively worse prospects is a way of mitigating simultaneous segments inequalities, and this is valuable in so far as one agrees with O’Neill or holds a similar view. This is what the forward-looking view tells us to do.

### The lifetime wellbeing view

A second reason to accept the forward-looking view can be inferred from the question of what a plausible *general* egalitarian or prioritarian theory might say. Both egalitarians who accept that inequalities in general wellbeing are unfair and prioritarians who believe that benefits to those who are worse off in terms of wellbeing matter more can combine their views with what Mathew Adler calls ‘nuanced’ notions of lifetime wellbeing that do not rely on additivity and atomism (Adler 2012, Chap. 6). Such a theory of lifetime wellbeing can incorporate many concerns that have been raised against the complete lives view in the general literature on equality, priority and time.

In order to illustrate what sort of theoretical opportunities arise when one abandons additivity and atomism, consider first the following outcome in which T1, T2, T3 are different successive periods of time with equal length, A, B and C are different individuals, and the numbers represent the invariable attributes (e.g. health) to wellbeing an individual has at a certain period of time. Assume that besides these attributes, the lives of A, B and C are identical.

**Table:** 

	T1	T2	T3
A	10	20	30
B	20	20	20
C	30	20	10

In case additivity and atomism hold, A, B and C are equally well off on the complete lives view (the attributes contribute with 60 to each of their lifetime wellbeing). If we abandon additivity so that attributes at different segments are treated differently depending on attributes at other segments, this does not have to be true. It could, for example, be that C is worse off than B, and B is worse off than A in terms of lifetime wellbeing. A rationale for aggregating attributes so that that follows might be that it is better to have a life that improves than a life that has an invariable level of attributes (this would explain why A is better than B), and better to have an invariable level of attributes throughout ones life than to have decreasing amounts of attributes (this would explain why B is better than C).

Furthermore, abandoning atomism allows us to say that A is actually better off in the first of these outcomes:

**Table:** 

Outcome X			
	T1	T2	T3
A	10	20	30
B	5	10	15

**Table:** 

Outcome Y			
	T1	T2	T3
A	10	20	30
B′	20	20	20

At each period of time, A has the same amount of attributes, and A’s life seems identical in the both outcomes. But if one abandons atomism, one can take into account how well off B is when one establishes how well off A is over her whole life. It might be better for A to be better off than B both at various times and over their complete lives, and perhaps this should be taken into account when one establishes how well off A is over her whole life.

One quickly realizes that abandoning additivity and atomism allows one to develop very refined approaches to individual lifetime wellbeing and how different attributes such as health contributes to an individual’s wellbeing. These can incorporate segment inequalities between persons, but also intrapersonal inequalities and the general distribution of goods over a life.

How does this relate to health inequalities and the question of how to establish who is worse off so that priority can be given to benefits to her when health is distributed? In the following way: one way of establishing who is worse off is by looking at who is worse off according to the general egalitarian or prioritarian theory that one embraces. Many such theories apply a complete lives view (cf. Adler 2012; Holtug 2010; Segall 2016). In case how good a life on the whole is established with a refined notion of individual wellbeing, it might well be that the forward-looking view toward health distribution is the best approximation of the impact of health distributions on lifetime wellbeing. The forward-looking view might well be something that the correct theory of individual wellbeing incorporates.

Settling what the correct theory of individual wellbeing is is of course beyond the scope of this paper. Here, I will mention two reasons for why the forward-looking view might plausibly capture something that is incorporated into a general approach to individual wellbeing. First, one consideration that might plausibly be important when we establish how good a life is is that it is better to lead a life that improves than to lead a life that becomes worse with time (cf. Velleman 1991). The reason for this might be that it is better for people to go through hardships in the beginning of their lives because that typically means that they are not used to leading a good life, and the hardships might mean that they will also appreciate later enjoyments more. Similarly, experiencing hardship late in life might be relatively worse since this typically means that one has a standard to compare with that is higher. If that is the case, being worse off at the beginning of ones life would count for less than being worse off at the end of ones life when lifetime wellbeing is established. This is not to say that it is not horrible to be badly off at a young age, but that the disvalue that 5 years of illness should be ascribed when a complete life is evaluated differs. A life with 5 years of illness during childhood followed by 40 years of perfect health might be better than a life with 40 years of perfect health followed by 5 years of illness.

Second, whether someone is worse off than others at specific temporal segments might affect lifetime wellbeing. One reason for this might be that simultaneous segment inequalities lead to inequalities in status goods like the ones that O’Neill points toward. Yet, it might also relate to other aspects of inequality. Being worse off than others at a specific time might affect for example what opportunities one has because the general structure of society tend to adapt to majorities. If that is the case, being worse off than others at a specific time will be taken into account by the correct theory of lifetime wellbeing.

If the correct theory of individual wellbeing over a life incorporates features like segment inequalities and the distribution of goods over a life, there are egalitarian and prioritarian reasons to mitigate segment inequalities in health, and to take these into account when priority is ascribed. A general egalitarian or prioritarian approach that relies on a refined notion of lifetime wellbeing provides reason to mitigate segment inequalities of specific goods, such as health. Considering the fact that past segment inequalities cannot be mitigated, this gives us some reason to apply the forward-looking view.

I believe that the arguments presented above at the very least show that the forward-looking view can be defended in a variety of ways, and I believe that they place the ball in the proponents of the complete lives view’s corner. It is up to those who defend the complete lives view to show why their view exhausts that which matters when we establish who is worse off with the purpose of assigning priority to different health benefits.

## Combining the complete lives view and the forward-looking view

How should the complete lives view and the forward-looking view be put together? In case the argument above is valid, this issue arises as an additional difficult aggregation problem that proponents of priority to the worse off have to engage with. So far, I have mainly introduced what I take to be easy cases in order to evoke different intuitions. But not all cases are easy of course. Consider, for example, the following distribution of health:

**Table:** 

Case 3					
Years	0–20	21–40	*t*_*0*_	41–60	61–80
Charles	0.3	0.5	|	0.6	0.4
Eric	1	1	|	0.5	0.3

Is Charles worse off than Eric so that benefits to him should be given priority? On the complete lives view, it is obvious that Charles is worse off. In terms of QALY, Charles enjoys 0.3 × 20 + 0.5 × 20 + 0.6 × 20 + 0.4 × 20 = 36 QALYs, while Eric enjoys 1 × 20 + 1 × 20 + 0.5 × 20 + 0.3 × 20 = 56 QALYs. In other words, Eric enjoys the equivalent of 20 years of life at perfect health more than Charles. Clearly, Eric is much better off than Charles in terms of health on the complete lives view.

However, in case we find ourselves at *t*_*0*_ and can provide some benefit to either Charles or Eric, it is not obvious that Charles should be considered to be worse off than Eric in the sense that implies that benefits to Charles matter more. On the forward-looking view, Eric is worse off than Charles: 0.6 × 20 + 0.4 × 20 = 20 QALYs, while 0.5 × 20 + 0.3 × 20 = 16 QALYs. On this view, it is Charles who has 4 more QALYs to enjoy, and Eric is much worse off than Charles.

In case we adopt a pluralistic approach to the issue of how to establish who is worse off when individuals are differently well off at different times, which I have argued for above, we need to balance the different views in cases such as this. How should we do this? Technically speaking, there is a variety of ways in which the two views can be combined. The plainest approach would be to simply add them up. Eric enjoys 20 QALYs more than Charles on one view, and Charles enjoys 4 more QALYs than Eric on the other view. 20 − 6 = 16, so perhaps we should say that taking both views into account, Eric enjoys 16 QALYs more than Charles and is thus better off.

Alternatively, one might ascribe lexical priority to one view. Perhaps we should always first look at the forward-looking view, and only invoke the complete lives view in case the forward-looking view fails to determine who is worse off. Or perhaps we should give the complete lives view lexical priority. Giving one view lexical priority is easy. However, lexical priority is also problematic. In particular, it is hard to explain why the threshold that is needed in order to establish exactly when one view becomes relevant should have such a large importance (cf. Arrhenius 2005; Arrhenius and Rabinowicz 2015). Why is it exactly at the point when a view cannot identify someone who is worse off that the other view becomes relevant?

What does strong priority mean? Generally speaking, it means that the forward-looking view matters more than the complete lives view, and that in case the different views provide different recommendations, the reasons to follow the recommendations of the complete lives view must be relatively much stronger than the reasons to follow the recommendations of the forward-looking view for us to have overall reason to follow the complete lives view. For example, in case the health inequalities on the complete lives view are very large, while they are small on the forward-looking view, we should follow the complete lives view. I do not know exactly how much stronger the reasons need to be, and since I generally believe that the normative realm is riddled by indeterminacy I suspect that we might never be able to determine this (cf. Herlitz 2016, 2017a, c, 2018b).

It might be objected that the pluralistic view that has been suggested in this paper is impractical, and thereby should be rejected in favor of approaches that are actually possible to use in health policy. There can be no doubt that things become more complicated when we accept the relevance of multiple dimensions, and sometimes this sort of complication entails big practical problems. Perhaps these practical problems are so overwhelming so that it is better to embrace a different approach.

I do not believe that this kind of objection gives us reason to adopt only the complete lives view, or only the forward-looking view. First of all, it is not particularly difficult to develop a model where one view takes lexical priority and the other view only works as a tiebreaker. That alone would be preferable to embracing only one view. Secondly, it is not so hard to combine the two views in other ways either. One way of dealing with the aggregation problem in practice which might appeal to health economists and others who value practicality, feasibility and usability would be to simply double count QALYs in the future, as explained above (add one view to the other). Thus, for example one could agree with the general suggestion of Ottersen et al. and apply Lifetime QALY prioritarianism (i.e. maximize QALY but give extra weight to benefits to those with fewer lifetime QALYs) but adjust this so that the priority weights depend on *both* lifetime QALY and the expected future QALY (Ottersen et al. 2014). This would at least capture the fact that both the complete lives view and the forward-looking view matter. In order to give strong priority to the forward-looking view, one could easily attach weights to the forward-looking view so that it has a larger impact on the overall assessment. This is of course a very coarse approach, but it is not particularly difficult to implement as long as one has the relevant data (which is a prerequisite for all approaches that have been suggested in the literature).

I favor the idea that we ought to give strong, but not lexical, priority to the forward-looking view so that in most instances when these conflict, we follow the recommendations of the forward-looking view and give priority to benefits to the worse off from that perspective. The reason why I favor this type of approach is that I believe that both of the arguments in the previous section have significant merit. I believe that segment inequalities in health matter, and I believe that they matter a lot. They entail differences in status goods, undermine self-respect, create undesirable power-relations and stand in the way of fraternal relations. I also believe there are good reasons to believe that a comprehensive approach to individual wellbeing needs to incorporate the fact that both the distribution of health over a life and health inequalities in temporal segments matter for individual wellbeing so that the forward-looking view gets support from egalitarian and prioritarian theories of distributive ethics. All of these considerations speak in favor of the forward-looking view, while the only reason I can see to accept the complete lives view is that it is unfair if different people have different amounts of health in their lives. I recognize that this matters, but on the whole I think it matters less than the reasons to embrace the forward-looking view. Thus, I believe that we ought to give strong priority to the forward-looking view.

## Discussion

In this paper, I have presented some challenges for the complete lives view toward how to establish who is worse off when we give priority to certain health benefits. I have not argued that this view is completely mistaken. I believe that the focus on complete lives has been beneficial in that it is a step away from a complete focus on current distributions of health. However, I think that the arguments presented in this paper give us reason to adopt a more nuanced approach to how to rank individuals in terms of who is worse off with the purpose of giving priority to certain benefits in light of unequal distributions of health over time. Such an approach accepts that both the complete lives view and the forward-looking view that only takes into account current and future health states matter. This leads to the complicated question of how to combine these views. Some work that addresses how to combine concerns for simultaneous segment inequality and complete lives inequality has appeared recently, but the question needs further attention, both by researchers who focus on the general value of equality and by researchers who focus on how to incorporate egalitarian considerations when health-related resources are allocated (cf. Bidadanure 2016; Davies 2016; McKerlie 2012). I presented my own view on this issue, that we ought to give strong priority to the forward-looking view, and tied this to the reasons for why the forward-looking view is important.

The practical implications of embracing the view that I have proposed are wide-ranging. Countries, international organizations, insurance companies and also individual altruists that decide how to allocate some of their scarce health-related resources by using cost-effectiveness analysis with the purpose of maximizing priority-weighted good health should, for example, change the way in which they ground the priority weights if they accept my view. Instead of grounding these weights in how badly off individuals currently are or in how much ill health individuals have over their whole lives, they ought to, on my view, ground these weights in a way that gives more importance to predicted future ill health. This implies, for instance, that conditions that can be predicted to cause relatively more problems in the future will be given greater priority.

There is a range of questions that need further attention in relation to how to establish who is worse off when people are differently well off at different times. Most obviously, the question of how to put the complete lives view and the forward-looking view together needs to be examined in further detail. Furthermore, increased attention should be given to the question of why it is important to give priority to the worse off. A better understanding of this will make it easier to establish who the worse off actually are. Yet, more work is also needed concerning how to spell out the details of the forward-looking view. A particular problem which I have sidestepped in this paper but that must be addressed concerns how to treat and delineate the relevant temporal segments (cf. Lippert-Rasmussen 2003; Segall 2016). Should we ascribe equal weight to each period of time in the future? How should we categorize future time periods? Answers to these questions will have vast implications for what the forward-looking view implies.

Accepting a view that takes into account how well off individuals are in the future and in the past also actualizes practical problems. Is it possible to predict people’s future health status? How should this be done? This will plausibly depend on the circumstances. Certain illnesses have predictable trajectories, while others do not. More research is needed on how to predict or estimate future ill health, but this is as big a problem for proponents of the complete lives view as it is for those who accept my proposal.

A different issue that I have deliberately avoided throughout the discussion above relates to age differences and differences in length of life and life expectancy. The forward-looking view seems desirable when we consider cases in which the different individuals are born at the same time and will lead lives of similar length. The intuitive appeal of this view might well change when we consider cases where individuals lead lives of different length and have different ages. Although some literature engages with these issues, how to take age differences and differences in length of life into account when we evaluate who is worse off is largely an unsettled issue (cf. Bognar 2008, 2015; Nord 2005; Williams 1997). This issue needs further attention, both in general moral and political philosophy and in population-level bioethics and health economics. It is my hope that the discussion in this paper can be of some use in research on this issue.

## Footnotes

1 It is not obvious to me what the authors mean by ‘worse off over their *lifetime*’. On one reading, they merely want to suggest that we must take all periods of a life into account when we assess who is worse off. On a different reading, they want to say that health-levels at all periods of time should be given *equal weight* when we assess who is worse off. The latter reading is in line with Williams’s suggestion, and also with the suggestion of Ottersen et al. The fact that Ottersen and Norheim are the (only) lead authors of the WHO report lends support to this latter reading. Yet, it might also have been left vague deliberately. The fact that the report strongly emphasizes how different countries must themselves engage with how to trade off different normative considerations lends some support to that interpretation.
